# Factors that influence patient empowerment in inpatient chronic care: early thoughts on a diabetes care intervention in South Africa

**DOI:** 10.1186/s12902-019-0465-1

**Published:** 2019-12-05

**Authors:** Nina Abrahams, Lucy Gilson, Naomi S. Levitt, Joel A. Dave

**Affiliations:** 1 Division of Health Policy and Systems, Department of Public Health and Family Medicine, University of Cape Town, Cape Town, 7925 South Africa; 2 Department of Global Health and Development, London School of Hygiene and Tropical Medicine, WC1E 7HT, London, UK; 3 Division of Endocrinology, Department of Medicine, University of Cape Town, Cape Town, 7925 South Africa

**Keywords:** Non-communicable disease, Inpatient, empowerment, Self-management, Diabetes, South Africa, Qualitative methods

## Abstract

**Background:**

The burden of non-communicable diseases is growing rapidly in low- and middle-income countries. Research suggests that health interventions that aim to improve patient self-management and empower patients to care actively for their disease will improve health outcomes over the long-term. There is, however, a gap in the literature about the potential role of the inpatient setting in supporting chronic care. This is particularly important in low-and-middle income countries where hospitals may be a rare prolonged point of contact between patient and health provider. The aim of this small scale, exploratory study was to understand what factors within the inpatient setting may affect patients’ feelings of empowerment in relation to their chronic disease care and provides recommendations for future inpatient-based interventions to support self-management of disease.

**Methods:**

This study was based in a public, academic hospital in South Africa. Eighteen qualitative, semi-structured interviews were conducted with multiple participants with experience of diabetes care: inpatients and health professionals such as nurses, endocrinologists, and dieticians. Findings were analysed using a broad, exploratory, thematic approach, guided by self-management and chronic care literature.

**Results:**

Interviews with both patients and providers suggest that patients living in low socio-economic contexts are likely to struggle to access appropriate healthcare information and services, and may often have financial and emotional priorities that take precedence over their chronic illness. Younger people may also be more dependent on their family and community, giving them less ability to take control of their disease care and lifestyle. In addition, hospital care remains bound by an acute care model; and the inpatient setting of focus is characterised by perceived staff shortages and ineffective communication that undermine the implementation of patient empowerment-focused interventions.

**Conclusions:**

Patient and provider contexts are likely to make supporting patient engagement in long-term chronic care difficult in lower income settings. However, knowledge of these factors can be harnessed to improve chronic care interventions in South Africa and other similar countries.

## Background

Estimates predict that by 2030 non-communicable diseases (NCDs) will account for five times as many deaths as infectious diseases in low- and middle-income countries (LMICs) and that 80% of global death from NCDs will occur in these countries [1, 2]. Compared to 23 other LMICs, South Africa (SA) has the third highest death rate due to NCDs per 100,000 adults – with cardiovascular disease and diabetes serving the largest load [3]. Beyond reduced quality of life, families face long-term medical expenses and loss of income earners, employers experience increased staff turnover and absenteeism, and it is estimated that between 2006 and 2015 diabetes, stroke, and coronary heart disease alone cost SA nearly two million US dollars in gross domestic product losses [1]. All of this is occurring in a country characterised by a quadruple burden of disease including NCDs, infectious diseases, injuries, HIV/AIDS, and a demographic shift resulting in people living for longer [4]. This disease profile of SA puts immense pressure on its already under resourced and overburdened health care system.

With this in mind, a large public hospital in South Africa sought to design and implement a Stewardship Programme that would better support growing numbers of patients presenting with diabetes complications in order to reduce the need for health services long-term health. International research into chronic care strategies highlights self-management and patient empowerment as important elements of effective chronic care as people need to manage their disease long after visiting a health professional [5–10]. While there is no set definition of empowerment, research tends to converge around measures of having control, being able to make decisions, believing in oneself, and being able to self-manage the disease [11]. An empowered patient would therefore be more able to adapt, question, challenge, and change their daily practices in an attempt to maximise their physical, emotional, and social wellbeing [12].

Currently, there is little application of chronic care and empowerment building models in hospital settings. A recent systematic review of self-management interventions for people with diabetes found positive health outcomes for interventions that engaged, educated, and motivated patients to set health goals and manage their insulin while in hospital [13]. However, only ten inpatient-based studies were identified and all were conducted in high-income countries. A mixed-methods analysis of over fifty of the top performing hospitals in the United States found they all had a high commitment to multi-disciplinary teams continuously engaging with patients about their needs and informing them about processes and future care while hospitalised [14], Hospitalisation is a prolonged point of contact between health professionals and patients that could potentially be used for patient education and training [15, 16]. Since many patients in this South African hospital come from a background of poor education and poor access to healthcare, the prolonged hospital stay represents a window of opportunity to make a significant impact on the ability of the patient to participate in self-care and become empowered. This is particularly important in LMICs where on discharge a person’s next access to a healthcare facility may only be after many months [17]. Therefore there is value in exploring the potential, but as yet, unclear, role that chronic care thinking can play in the unique setting of the hospital, particularly in a low-income and high NCD burden context.

This paper reports exploratory research undertaken to support the development of this hospital intervention. The aim of the research was to understand what factors in patients’ personal lives and in the health system influence whether patients feel empowered to self-manage their chronic disease once they leave a hospital setting. The paper draws out lessons for future inpatient empowerment interventions to consider in their design and implementation.

## Methods

### Setting, sampling and data collection

This study was conducted in a tertiary South African public hospital in early 2018 prior to the planned introduction of a patient empowerment intervention. Eighteen participants were purposively sampled based on whether they had recently participated in providing in-patient healthcare to patients with diabetes or whether they were a patient with diabetes that had recently been admitted to the hospital. Participants were then grouped into three categories: six health professionals, including endocrinologists, dieticians and a pharmacist, who were in charge of conceptualising, designing and implementing the planned Diabetes Stewardship Programme (A), five health professionals, namely nurses, who had the most contact time with patients with diabetes in the hospital (B), and seven people who have diabetes and had recently experienced being an inpatient in the hospital (C). Thirteen participants were female but all ranged in age, and time spent at the hospital.

Using the established selection criteria, two endocrinologists from the hospital’s Division of Endocrinology (NL, JD) identified initial potential participants who were then asked to suggest further participants in order to include a relevant mix of stakeholders. Potential participants were either emailed (if their email address was available) or contacted by the primary researcher whilst in the hospital to discuss participating in the study. Information was given about the primary researcher (NTA, female MPH researcher), the research team (first and second authors as outside evaluators, third and fourth authors part of endocrine team at hospital) the proposed intervention, the aim of the study, and what the participant can expect from the interview. If they agreed, a time was set up for the interview in a location in the hospital that suited the participant. Only two potential participants refused to participate in the study due to feeling too ill (patient) or being too busy (health professional). For this small exploratory study, data saturation was considered complete once the authors or participants did not suggest anyone new that should be included.

At the agreed upon date and time informed consent was obtained and one-on-one in-depth interviews were conducted between the primary researcher (NTA, a qualitative health policy and systems researcher) and the participant (with the addition of a translator for two patients). No other hospital staff or patients were present during the interview time. Interviews were semi-structured using questions, developed by the primary researcher based on wider literature on chronic care and empowerment and approved by the research team. While not formally piloted, these questions were further refined for relevance after the first few interviews were conducted in order to elicit the experiences and thoughts of respondents on current inpatient practices and chronic care. Questions included open-ended exploratory inquiries such as “what is the Diabetes Stewardship Programme and why would you like to implement it?” What factors make it difficult or easy to implement new interventions?” “How would you describe an empowered patient?” “What difficulties do nurses experience when interacting with patients and new interventions?” “What was your experience of the inpatient care and how can it be improved? “What factors in your personal life make it difficult to manage your diabetes?” During interviews, participants were asked their opinion on issues raised by other participants in order to cross-validate information and increase rigour of the findings [18, 19]. The primary researcher also made simple reminders and notes during the interviews to document thoughts about potential themes in real-time. Sixteen of the interviews were conducted in English and two in isiXhosa using a translator. Interviews varied in length between 20 min and one hour. Interviews were recorded, transcribed and anonymised by the primary researcher.

### Data analysis

Given limited existing experience of hospital-based diabetes patient empowerment programmes in LMICs, the research took a broad exploratory thematic approach useful in finding out what is happening in little understood situations [18]. Guided by patient empowerment literature, the primary researcher identified possible themes and codes within the interview transcripts. These were hand coded as the sample was relatively small. The second author (LG, a qualitative health policy and systems researcher) then compared a subset of transcripts to themes identified in order to validate and strengthen the rigour of the analytic process [18]. Considering the small scale of the study and limited literature on hospital chronic care, the analysis was focussed on gaining an initial picture of the contexts that explain patients’ ability to feel sufficiently empowered to care actively for their chronic disease. Themes related to current empowerment literature or raised by multiple participants were included. Quotes that represented the theme in a concise and effective way were chosen by the primary researcher and discussed and agreed upon by all the authors.

## Results

The experiences of participants were categorised into three broad themes: patient context, the inpatient setting, and the patient-provider relationship.

### Patient context

Five of the patients interviewed were young, in their 20s or early 30s, and had been diagnosed with diabetes within the last month; the other two were in their 40s and had been living with diabetes for over ten years. The participants had a mix of Type 1 and Type 2 diabetes and were hospitalised for a variety of reasons. The younger participants were hospitalised for symptoms relating to diabetes such as onset of symptoms for the first time (short breath, frequent urination, weight loss, increased thirst) or similar symptoms due to not taking their medication correctly. One of the older patients was hospitalised for a leg amputation due to his diabetes while the other was hospitalised due to a new diagnosis of cancer.

All patients came from lower income communities. The wider context of poverty, and its impact on family and friends, clearly influenced patients’ lifestyles, and their approach to chronic health care and health professionals’ advice. Firstly, all patients had family or friends with diabetes and so, while the newly diagnosed patients were often nervous about the disease, they were still confident that they understood it. However, the advice and support offered by others was often misinformed and ineffective, as their peers did not always have access to the best sources of health education and resources in their LMIC setting. This led, sometimes, to patients engaging in inappropriate care or being discouraged from seeking formal health care.

“*At the beginning I was not that confident because people were saying you are going to be cut, so I was like okay let me just keep my mouth shut … Yes, sometimes, like negative. ‘It’s better to have HIV than [diabetes]’... I feel like I’ve got this disease that is normally in the old people, now I’ve got this disease like I did something wrong, what did I do now?” [C2].*

In addition, if the patients did become aware of the misinformation surrounding them, they often felt unable to access appropriate information and care as clinics were far away, medication expensive, or health providers in their communities were perceived to be of relatively low quality. Only one participant had a job; two had to resign from their jobs when admitted as inpatients, and the others relied on family support or a social grant to pay for health care appointments, transport, and medical equipment. This meant that health care was often not a priority compared to other financial demands.

“*The two main issues that stops [patients] engaging in primary care. The first one is their social situation, there are far more important things in their life like ‘where do I get my next job from, my son is a drug addict, there’s no food on the table, what am I going to do?’” [A2].*

*“Food insecurity is a huge issue... Crime huge issue, their monitor is being stolen, their medication is being stolen, being mugged, going to the day hospital is often dangerous. So it’s a huge challenge.” [A6].*

The availability of financial and economic support affected, therefore, the extent to which patients with a chronic illness were able to prioritise caring for their health over other needs.

*“A very empowered patient, if there’s nothing that you’ve got to eat and at the second day of not eating you get given a chocolate you eat the chocolate, it doesn’t matter how empowered you are.” [A6].*

The setting of low socio-economic status (SES), misinformation, and inadequate healthcare access significantly affected the younger patients. This group all still lived with their immediate family – likely due to financial dependence and social norms - and so were all influenced by family norms more than the older, independent patients. They mentioned the challenge of their family eating unhealthy foods, and how this made it more difficult to adhere to their diet plans. Three patients (and two health professionals) noted that young patients did not like feeling different from others, as when eating different foods and pricking their finger, or feeling like they cannot engage in fun but unhealthy activities with their peers. In contrast, one of the older patients, who headed her household and had more independence, income and experience, was better able to influence her family to engage in healthier lifestyle activities, making it easier for her.

Health professionals and patients both reported that an additional burden for young people in this setting could be a sense of helplessness over future financial stability and potential employment.

*“In the young adult clinic there’s a sense of hopelessness about jobs. They sit around at home, they are isolated they are alienated they don’t have friends in the neighbourhood. It’s a really hard actually. We need much more social support. It’s a lonely business.” [A5].*

These frustrations often then presented themselves in defiant and dismissive behaviour towards health care. Two health care professionals noted that younger people were less compliant with authority than other groups, took more health risks, and had less commitment to long-term care.

*“But the thing is mostly young people, in their teens and early 20s. And they are the group that’s usually unfortunately uncompliant. They do not take medication, they do not rock up for appointments, they always have one or another excuse.” [B1].*

The dependence of young patients on families also added complexity to their health care engagement. Two health professionals emphasised the importance of including family in the diabetes education of the patient – in order to increase household knowledge and so develop patient support systems. However, another health professional warned that some families can try to take control of appointments, assuming patient naivety and dependence, discouraging young patients from taking an active role in their care. Patients may, then, be tempted not to share their diabetes status with others in fear of being reprimanded when ‘cheating’ on their diet or lifestyle.

*“[The patient] refuse to come to the clinic because [the doctors] have access to their mother at home, and their mother constantly when he comes here will reprimand him and he couldn’t handle it.” [B1].*

Despite the challenges, patients were also sometimes motivated to engage in their chronic care. All the young patients mentioned that being surrounded by appropriate support systems was helpful. Two patients commented on the usefulness of local diabetes clubs for education, advice, and medical care access. Four of the patients drew motivation from their children as they wanted to be healthy and see them grow. One health professional mentioned that when patients had high levels of resilience then they were able to overcome many of their health care barriers.

*“So there’s the person and then there’s where they live and it’s the interaction between the two, and there’s resilience and there’s ability to cope, there’s support. They all play out differently.” [A5].*

Three of the patients mentioned that the shock of experiencing or seeing adverse symptoms, especially those that resulted in hospitalisation, helped them understand how serious their diagnosis was and how necessary it was to heed health advice.

### The inpatient setting

The hospital setting, providing short-term inpatient care for sick patients, offers a particular set of circumstances that affects the way health providers are able to engage with patients and vice-versa.

Patients are particularly sick when hospitalised, which affects their care in various ways. Firstly, three patients noted that the day-to-day experience in the hospital did not reflect real world behaviours: receiving all meals ready-made and eating when it suited the hospital; undergoing intense medical procedures; and the difficulty of sleeping amongst tubes and hospital sounds.

“*Of course when patients are admitted to the ward they are completely disempowered. That’s how hospitals work. You take all their rights and privileges away. You even take their clothes away and give them a funny white blue gown where their bum sticks out. You know they are completely disempowered and they have almost no say in what happens to them.” [A2].*

Other patients also noted that they were distracted by how they were going to afford their health care costs, especially when they were unable to work while hospitalised.

Secondly, four health professionals perceived that the hospital care was fragmented, despite high rates of multi-morbidity in chronic disease. Initial treatment is provided for the problem for which the patient is admitted, making it difficult for health providers to respond holistically. One health professional explained that doctors in different wards can even forget to record diabetes medicine when discharging patients, forcing patients to return prematurely in order to retrieve the correct medication.

*“It is a problem because the way the system is designed when you’re in a surgical ward for procedures you’re being managed for the procedure, so diabetes is not your focus … we’re focusing now on your procedure I’ll just continue whatever was done even if it’s not appropriate.” [A1].*

Another feature of the inpatient setting is the acute nature of care. Providers found that in the rush to treat a patient there was little time to provide relevant education and allow for patient independence in their care, even if the patient was relatively healthy and had time.

*“There is a lot of potential contact hours, other than the 6 minutes and [the doctor is] gone. [The patient is] in the hospital for many many hours, 5 days 6 days, so there’s lots of opportunity for contact time”. [A2].*

One nurse mentioned that as responsibility rested with her if something went wrong, it was easier to take control and guarantee that the patients were receiving the necessary treatment.

*“It’s inpatient, so the patient is totally dependent on us as nursing staff. We do the blood glucose checking, insulin we give ourselves. We had times when people were taking their own insulin but their glucose don’t come down or they just shoot up. They don’t do the insulin correctly so the nursing staff we do everything for the patient, as an inpatient the patient is our responsibility … The insulin is kept in the fridge and the needles is kept with us, you know with medical and legal hazards so it’s better if we give it.” [B3].*

### Shortage of staff

The perceived shortage of staff was also noted as encouraging provider control over patients, as health professionals found they did not have the time to train, watch, and retrain patients in their own care.

“*What she also mentioned to me, which I wasn’t happy with, I asked ‘do you tell the doctor all these things?’ she said ‘I would have but the doctor was in a hurry’”. [B1].*

All the nurses explained that not being able to provide quality care due to staff shortages and long hours left them feeling tired and demotivated to work. They could not then gain the gratification of seeing patients improve, nor have the energy to lobby for better hospital processes or attend extra training workshops. As the frontline workers who interact most with patients they were expected to take full responsibility for all aspects of patient care – beyond levels they felt were reasonable given the hospital constraints.

“*Six nurses for a whole ward of 30 patients, with walls in between. We can’t see behind that wall what that patient is doing, but management asks ‘where are the nurses?’ So six nurses must now cut themselves, put my arm there, my head there then at least every part will look at the patients.” [B3].*

As the hospital under review serves as a training institution, it had particularly high levels of staff turnover. Health professionals were often on training rotation, support from medical students disappeared during examinations, and graduates often moved on.

### Health system bureaucracy

Health providers were also influenced in many, less obvious, ways by the bureaucracy, institutional habits, and relationships that occur in complex health systems. For example, all the health professionals experienced problems with the ways in which information or expectations from leadership was shared, and how changing information often created confusion and so resistance. Specifically, respondents noted that older providers often become ‘*stuck in habits’ [A6]*, and become wary of any new guidelines that may add to their workload. In addition, most health professionals indicated that the short-term staff hired to make up for shortages were often disinterested in new protocols making it difficult to create teams with good communication and updated training.

*“The first thing is getting buy-in from everybody, making sure everyone is educated. It’s actually quite hard, partly because the staff rotate all the time. Our nursing staff, 40% are locum staff, they don’t belong to the hospital, they don’t care about the hospital, they just here to do a shift and then leave, they don’t have a clue what’s going on... The doctors rotate every three months, depends on where you catch them. So you have this constant staff turnover.” [A2].*

*“…the cogs of the wheel in this huge bureaucracy move very slowly.” [A6].*

One important, but difficult and time-consuming, strategy that all of the of health professionals noted would support the implementation of improvement strategies is that of prioritising staff buy-in. Providers suggested this would call for all staff to be consulted and engaged with plans for change before they took place. They felt that this would not only help to identify the potential problems of the proposed programme before implementation but would also encourage staff to understand and proactively engage in implementing new interventions.

*“I think sometimes the resistance is always from staff initially. Is it added work for us? How are we going to fit this into an already tight schedule? And usually the resistance decreases when you can see the impact.” [A1].*

It is important to note however that the key concern is *how* staff are engaged rather than just engaging widely. For example, five health professionals felt that workshops and personal engagement were more useful than receiving a long text about changes. In addition, all providers noted that staff need to feel that they are being included in changes and that their opinion is actually truly valued.

*“And they will see that the people that’s doing the work, like us here, we will come up with the right solutions because we deal with it on a daily basis and not management upstairs.” [B1].*

Insubstantial engagement on the other hand could lead to continued resistance to new hospital processes.

*“Communication is good. But sometimes communication is very bad amongst staff … doctors included. And the higher authority there. They just come on us and dump things on us ‘dwah’. Like we are robots. We are not robots, we are also human beings but to them we are just a workforce… Not to say that we don’t have a say but when it comes to us it’s already decided …. They just give orders, we’re the followers … some of them don’t even greet you, they just greet you if they want something.” [B3].*

### Patient-provider relationship

The context of the patient and the health provider interacted in complex ways. Three respondents recognised that the controlling nature of the inpatient setting, coupled with staff-shortage and turnover issues, meant that health professionals could often become inflexible, rushed, and rude to patients. This could, in turn, create resistance from patients, as they felt they were not being given attention nor offered holistic care. One patient relayed her experience of dealing with a doctor who argued that he was the health expert and so would not listen to her.

*“He said okay I give up. Before he said that he pointed his finger at me and said ‘as long as you are in this hospital you are not going to refuse this’ …. I am the one who is diabetic, I have experiences… This is my body; I’ve been diabetic for 20 years. I know what is right and what is wrong. I may not be a doctor but I know about diabetes, I learn in in the clinic about diabetes, I go to clubs. So I know what I’m talking about.” [C7].*

Three of the health professionals noted that the low SES context of the patient greatly affected the way they related to them. One provider explained that some patients sold their medication and equipment, as they were desperate for the finances. Another admitted that providers engaged more actively with patients from particular age and income groups who were assumed to be more capable of taking care of their chronic disease.

“*I think we are sometimes judgemental if a student comes here in tertiary education then we give all the information. Now someone comes from a rural area then we give only certain information which is unfair. We should actually give all information to everybody, what they do with it is their problem, but we sometimes judge people when it comes to that.” [B1].*

While often easier for health professionals to take control, this “*treating patients like a baby” [B1]* approach, could result in negative effects. One health professional complained that in the time between discharge and follow up appointments, patients have often not correctly used their medicines because they were not adequately empowered during their inpatient stay. Another admitted that this controlling approach could dampen a patient’s ability and enthusiasm to do things for themselves, resulting in poor health outcomes post-discharge.

“*I think sometimes they don’t feel like going home, because sometimes they feel, not that they going to be neglected, but I mean here they being cared for, take the tablets, and washed. Now they scared to go home because they scared to keep up.” [B2].*

Two health professionals reported that they have played social worker in order to navigate the many needs of a patients and that this can be a heavy emotional burden for them. Considering this, they explained how necessary it was to work closely with other social welfare sectors.

*“When it comes to the hospital, it’s like we expect for them to give us all information which they are not giving. We want them to be open and honest but they’re just a human being. I had a patient that I must educate here, and when I said regarding the insulin ‘try with something to eat’. It’s no use I’m having all this pamphlets and magazines I’m showing her and then what she said sometimes she needs to go to the neighbour to get something to eat just so she can take her insulin. And I stopped the education immediately and … I referred her to Social Work for a food parcel. Because sometimes we educate and we educate but forget the circumstances at home.” [B1].*

Patients and health providers did however note some important roles that the hospital played in encouraging patient enthusiasm and engagement in chronic care. For example, as the hospital is better resourced than many other health facilities in the country, it could often better provide equipment and medicines to patients. Two patients relayed their experience of being able to access insulin and a glucose meter at the hospital and not elsewhere. Two health professionals explained that when they went beyond their expected hospital role and made a deeper connection with the patient, then they were able to overcome many of the barriers of the hospital and home context. Specifically, including patients in creation and planning of their treatment plan, ensuring they understand the disease and medical procedures, and providing a sense of security and understanding that encouraged honest and open patient-provider engagement.

*“You not only have to educate but also convince them to want to be educated … So then, from there I notice [I] have a bond with them… … If I am saying there is a need for this close relationship, it’s amazing because they get so interested, they come … So they feel so motivated because at least now they are participating…I am always telling them that I am with them in their journey… to be in partnership with them, they really like it.” [B5].*

## Discussion

NCD management and prevention is quickly becoming policy priority within global and local contexts in both high and low-income countries [20, 21]. With this rise, there is a wide range of literature on the effectiveness of chronic care models and the importance of fostering self-management and empowerment within people with chronic diseases in order to improve long-term health outcomes [7–9]. However, there is limited understanding of the potential role that acute care hospitals can play in supporting these chronic care goals, particularly in LMICs where hospitals may be a rare prolonged point of contact between patient and health provider. Considering the increasing burden of chronic illnesses within LMICs such as South Africa, this study aimed to explore what factors may support or hinder chronic care thinking within an LMIC inpatient setting using an appropriate qualitative study design. The study’s findings are useful in informing potential hospital interventions aimed to enhance patients’ ability to care actively for their chronic disease after hospitalisation.

### Patient empowerment levels moderated by patient factors

Patients are influenced by multiple factors that moderate their ability to engage with long term care. Research in LMICs indicates that factors such as social support availability, self-efficacy and the opportunity to make independent health care decisions, and disease condition contribute to perceived ability to care for one’s diabetes long-term [22–24].

In this South African study, young and dependent groups within a low SES setting in particular were often misinformed about chronic diseases and engaged in activities that did not support long-term health care. Global literature [23–25] supports the finding that chronic care patients from lower socio-economic backgrounds often experience barriers to accessing quality health care and often do not have the social and financial support to encourage long-term lifestyle changes.

Among the reasons explaining these findings in South Africa may be the high level of unemployment - nearly one third of the South African population is unemployed [26]. This not only affects ability to access quality care but also decreases a person’s subjective wellbeing, confidence in their abilities and leads to high levels of boredom, uncertainty and feelings of isolation [27–29]. In addition, the trend towards urbanisation in South Africa has particularly contributed to urbanised youth accessing cheap, processed foods, following sedentary lifestyles, and engaging in risk factors for NCD development [30, 31]. These systematic reasons contribute to the difficulty in accessing appropriate health care and maintaining care for one’s chronic disease over a long time. Comparatively, patients with greater resilience, support and independence are better able to navigate the barriers to caring for their chronic disease [25, 32].

### Patient empowerment levels moderated by provider and inpatient factors

The health system factors likely to influence patient experience can be understood as comprising both hardware and software factors [33–35]. The hardware of finances, infrastructure, medicine and technology availability, and staff availability affect the success of empowerment-focused interventions when resource constraints impede providers’ abilities to implement effective patient-centred strategies and motivate patients to care for their chronic illness [36]. Resource availability continues to be a major barrier in LMICs where funding for healthcare is largely allocated towards infectious diseases such as HIV and TB and little is directly earmarked for NCDs [2]. In addition, there is a lack of trained health workers and low retention of the health workforce. In South Africa, specifically, experience shows that there are high levels of inappropriate referrals and delayed diagnosis and care for those with chronic illnesses due to unavailability or inefficiency of services and staff [17].

A system’s software on the other hand contains tangible elements such as information use, skills development, decision-making processes, as well as intangible elements such as values, power, trust, and norms that govern actions between the people who work within the system, including providers and patients. This study found that the organisational culture, including leadership patterns and communication style, affected the potential success of empowerment-focused interventions. For example, centralised and hierarchical decision-making within health care is recognised as undermining providers’ and communities’ engagement in service delivery decision-making [37]. This has resulted in the development of inappropriate policies that are not well implemented, slowing down potential service delivery improvements [38]. In addition, leadership and decision-making strategies that do not adequately consider the needs and capabilities of middle managers and frontline workers can also result in dissatisfaction and lack of commitment from providers, who in turn moderate the effectiveness of empowerment-focused interventions [39].

Health professionals’ own personal values and professional goals can also affect their motivation to implement new interventions [36, 39]. This includes whether the provider feels competent to provide the interventions, whether the provider sees that they are able to attain the professional opportunities that they would like, as well as the openness and willingness of the provider to change, which can itself be mediated by factors such as level of training and age of provider [23, 24, 40, 41].

Health system research on barriers to NCD care is growing rapidly; however, is focused largely on preventative, primary care [42]. Therefore, this study specifically considered the role that inpatient care could potentially play in patient chronic care engagement. The study’s finding that patients may be too sick to engage in and retain information on long-term lifestyle change education within an inpatient setting is supported by wider experience [43]. In addition, the disease focus of hospital care can result in limited holistic care for patients and increased need for re-admission [44]. Within the LMIC inpatient setting, staff and bed shortages mean health providers are also under pressure to treat and discharge patients as quickly as possible, leading to increasingly controlling and rushed patient-provider interactions that hinder patient self-efficacy and learning [45]. Nonetheless, participants and wider literature note that hospitalisation is a prolonged point of contact between patients and health professionals – especially in LMICs - that, barring other constraints, could prove useful for patient-centred interventions [14, 16].

### The implications of study findings

This small scale and exploratory study, in combination with wider relevant literature, suggests that hospital-based interventions that aim to engage patients with chronic diseases in long-term care should consider relevant patient and health system factors that moderate levels of patient empowerment. This is particularly pertinent in an LMIC setting such as South Africa that has well-documented funding shortages that necessitate creative thinking for health system change [46].

For example, one participant suggested that, in order to foster holistic care within an acute care setting, hospitals could introduce disease ‘expos’ that bring together staff and patients from different wards in an informal way, and in a time that suits them, to learn about the similarities of causes and care between different acute and chronic diseases. Thought could also be given towards reducing the workload of health professionals in order to prevent staff burnout and demotivation, and allow providers more time to engage with patients [36, 45]. This might be achieved in part by simplifying and reducing administrative tasks or by task-shifting to other personnel [47]. Staff might also develop systems for identifying which patients are well enough to engage in their care based on common barriers found such as severity of illness, financial status, length of stay, and availability of staff in order to support patients administering their own medication while admitted; both empowering these patients and alleviating provider workloads [16, 43]. Improved communication amongst staff that speaks to providers’ goals could also help to motivate health professionals to engage in patient-centred interventions. This includes mutual constructive engagement, fostering trust as well as ensuring everyone understands the health system vision, feels included in intervention development, and can see the improvements that are a result of their work [8, 36, 48, 49]. Decreasing staff workload and increasing staff motivation and cohesiveness could potentially then create an opportunity to take advantage of the time patients are in the health system setting and implement programmes that increase self-efficacy and active learning [50]. Strengthening hospital relationships with discharge facilities as well as larger policy development organisations could also contribute to streamlining inpatient systems and including the inpatient setting in the growing NCD agenda [2, 21, 37]. These shifts could then contribute towards fostering a hospital environment that is able to support the development of chronic care intervention packages that take into consideration the context, age, and diagnosis status of the patient.

### Limitations

There are various limitations to this study. First, inpatients who were interviewed had a certain level of health and enthusiasm to participate that is not indicative of the entire inpatient population. Second, four of the seven patients interviewed had only recently been diagnosed with diabetes and experienced inpatient care. This made it difficult to gauge fully from these patients how they felt that inpatient care affected their feelings of empowerment in the long-term. Therefore, long-term inpatient care effects on patients’ feelings and behaviours were largely understood through the experiences of the healthcare providers. Despite these constraints, the study participants offered a wide range of experiences in diabetes inpatient care and the findings offer ideas that can be further considered over time in this, and other, health care settings.

This study was small scale and exploratory, and so offers insights for the hospital where it was conducted rather than more widely generalisable findings. The overall study design was, however, relevant to the research aim and, as discussed, the findings are comparable to the wider knowledge on this topic, suggesting they may have resonance in other South African and LMIC settings. The study was also conducted rigorously in terms of research practice relevant to this type of study. For example, interview questions were initially developed from review of relevant literature and adapted after initial experience. The data were, moreover, analysed following appropriate principles for thematic analysis, with coding involving two authors and final data interpretation involving all authors [18, 51].

## Conclusion

Multiple barriers and facilitators within the patient and health system context affect the ability of a patient to engage in long-term care for a chronic disease, and the ability of health facilities to implement patient empowerment interventions. Knowledge of these factors is important in developing strategies to improve patient empowerment. Specifically, this study suggests that it is not only tangible resources, such as finances and infrastructure, that are important in long-term health care but also less tangible elements such as communication, resilience, and trust. Responding to a gap in the literature, the study serves as a starting point for creative thinking about how the current South African health system can respond to the pandemic of NCDs using hospitals as a valuable window of opportunity for developing and implementing much needed patient empowerment strategies.

## Data Availability

The datasets used and/or analysed during the current study are available from the corresponding author on reasonable request.

## Abbreviations

LMICs: Low- and middle-income countries
NCDs: Non-communicable diseases
SA: South Africa
SES: Socio-economic status

## References

[1] Hofman K. Non-communicable diseases in South Africa: a challenge to economic development. S Afr Med J. 2014. 10.7196/SAMJ.8727. 10.7196/samj.8727 25363059

[2] Samb B, Desai N, Nishtar S, Mendis S, Bekedam H, Wright A, et al. Prevention and management of chronic disease: a litmus test for health-systems strengthening in low-income and middle-income countries. Lancet. 2010. 10.1016/S0140-6736(10)61353-0. 10.1016/S0140-6736(10)61353-0 21074253

[3] Alwan A, MacLean DR, Riley LM, d’Espaignet ET, Mathers CD, Stevens GA, et al. Monitoring and surveillance of chronic non-communicable diseases: progress and capacity in high-burden countries. Lancet. 2010. 10.1016/S0140-6736(10)61853-3. 10.1016/S0140-6736(10)61853-3 21074258

[4] Mayosi BM, Benatar SR. Health and health care in South Africa — 20 years after Mandela. N Engl J Med. 2014. 10.1056/NEJMsr1405012. 10.1056/NEJMsr1405012 25265493

[5] Anderson RM, Funnell MM. Patient empowerment: myths and misconceptions. Patient Educ Couns. 2010. 10.1016/j.pec.2009.07.025. 10.1016/j.pec.2009.07.025 PMC2879465 19682830

[6] Aujoulat I, d’Hoore W, Deccache A. Patient empowerment in theory and practice: polysemy or cacophony? Patient Educ Couns. 2006. 10.1016/j.pec.2006.09.008. 10.1016/j.pec.2006.09.008 17084059

[7] Bodenheimer T, Lorig K, Holman H, Grumbach K. Patient self-management of chronic disease in primary care. JAMA. 2002. 10.1001/jama.288.19.2469. 10.1001/jama.288.19.2469 12435261

[8] Davy C, Bleasel J, Liu H, Tchan M, Ponniah S, Brown A. Factors influencing the implementation of chronic care models: a systematic literature review. BMC Fam Pract. 2015. 10.1186/s12875-015-0319-5. 10.1186/s12875-015-0319-5 PMC4545323 26286614

[9] Epping-Jordan J, Pruitt S, Bengoa R, Wagner E. Improving the quality of health care for chronic conditions. Quality & Safety in Health Care. 2004. 10.1136/qshc.2004.010744. 10.1136/qshc.2004.010744 PMC1743863 15289634

[10] Norris SL, Engelgau MM, Venkat Narayan KM (2001). Effectiveness of self-management training in type 2 diabetes. Diabetes Care.

[11] McAllister M, Dunn G, Payne K, Davies L, Todd C. Patient empowerment: the need to consider it as a measurable patient-reported outcome for chronic conditions. BMC Health Serv Res. 2012. 10.1186/1472-6963-12-157. 10.1186/1472-6963-12-157 PMC3457855 22694747

[12] EMPATHiE Consortium. Empowering patients in the management of chronic diseases (final summary report). Health Programme of the European Union 2014. http://ec.europa.eu/health//sites/health/files/patient_safety/docs/empathie_frep_en.pdf. Accessed 14 Apr 2017.

[13] Taylor SJ, Pinnock H, Epiphaniou E, Pearce G, Parke HL, Schwappach A, Purushotham N, Jacob S, Griffiths CJ, Greenhalgh T, Sheikh A. A rapid synthesis of the evidence on interventions supporting self-management for people with long-term conditions: PRISMS–Practical systematic Review of Self-Management Support for long-term conditions 2014; doi: 10.3310/hsdr02530. 25642548

[14] Aboumatar HJ, Chang BH, Al Danaf J, Shaear M, Namuyinga R, Elumalai S, Marsteller JA, Pronovost PJ. Promising practices for achieving patient-centered hospital care 2015; doi: 10.1097/MLR.0000000000000396. 10.1097/MLR.0000000000000396 26147867

[15] Korytkowski MT, Koerbel GL, Kotagal L, Donihi A, DiNardo MM. Pilot trial of diabetes self-management education in the hospital setting. Primary Care Diabetes. 2014. 10.1016/j.pcd.2013.11.008. 10.1016/j.pcd.2013.11.008 24387916

[16] Maybrey ME, Setji TL. Patient self-management of diabetes care in the inpatient setting: pro. J Diabetes Sci Technol. 2015. 10.1177/1932296815590827. 10.1177/1932296815590827 PMC4667327 26092686

[17] Goudge J, Gilson L, Russell S, Gumede T, Mills A. Affordability, availability and acceptability barriers to health care for the chronically ill: longitudinal case studies from South Africa. BMC Health Serv Res. 2009. 10.1186/1472-6963-9-75. 10.1186/1472-6963-9-75 PMC2694171 19426533

[18] Gilson L. Doing health policy and systems research: Key steps in the process. In: Health policy and systems research: A methodology reader. World Health Organisation. 2012. http://www.who.int/alliance-hpsr/alliancehpsr_reader.pdf. Accessed 09 Nov 2016.

[19] Sobh R, Perry C. Research design and data analysis in realism research. Eur J Mark. 2006. 10.1108/03090560610702777.

[20] Horton R, Sargent J. 2018 must be the year for action against NCDs. Lancet. 2018. 10.1016/S0140-6736(18)30674-3. 10.1016/S0140-6736(18)30674-3 29627163

[21] Department of Health. Strategic plan for the prevention and control of non-communicable diseases 2013–17. https://extranet.who.int/ncdccs/Data/ZAF_B3_NCDs_STRAT_PLAN_1_29_1_3[2].pdf

[22] Peters DH, Garg A, Bloom G, Walker DG, Brieger WR, Rahman H. Poverty and access to health care in developing countries. Ann N Y Acad Sci. 2008. 10.1196/annals.1425.011. 10.1196/annals.1425.011 17954679

[23] Kardas P, Lewek P, Matyjaszczyk M. Determinants of patient adherence: a review of systematic reviews. Front Pharmacol. 2013. 10.3389/fphar.2013.00091. 10.3389/fphar.2013.00091 PMC3722478 23898295

[24] Mackian S, Bedri N, Lovel H. Up the garden path and over the edge: where might health-seeking behaviour take us? Health Policy Plan. 2004. 10.1093/heapol/czh017. 10.1093/heapol/czh017 15070862

[25] Bravo P, Edwards A, Barr PJ, Scholl I, Elwyn G, McAllister M, et al. Conceptualising patient empowerment: a mixed methods study. BMC Health Serv Res. 2015. 10.1186/s12913-015-0907-z. 10.1186/s12913-015-0907-z PMC4488113 26126998

[26] Moya S. South Africa unemployment rate. 2018. https://tradingeconomics.com/south-africa/unemployment-rate. Accessed 08 Jun 2018.

[27] De Witte H, Rothmann S, Jackson LTB. The psychological consequences of unemployment in South Africa. S Afr J Econ Manag Sci. 2012. 10.4102/sajems.v15i3.153.

[28] Di Tella R, MacCulloch RJ, Oswald AJ. Preferences over inflation and unemployment: evidence from surveys of happiness. Am Econ Rev. 2001. 10.1257/aer.91.1.335.

[29] Harnois G, Gabriel P. The importance of work to an individual’s mental health. In: Harnois G, Gabriel P, editors. Mental health and work: Impact, issues and good practices. Geneva: World Health Organisation; 2000. p. 5.

[30] Kruger HS, Puoane T, Senekal M, van der Merwe T. Obesity in South Africa: challenges for government and health professionals. Public Health Nutr. 2005. 10.1079/PHN2005785. 10.1079/phn2005785 16153330

[31] Peer N, Bradshaw D, Laubscher R, Steyn N, Steyn K. Urban–rural and gender differences in tobacco and alcohol use, diet and physical activity among young black south Africans between 1998 and 2003. Glob Health Action. 2013. 10.3402/gha.v6i0.19216. 10.3402/gha.v6i0.19216 PMC3559753 23364100

[32] Yi JP, Vitaliano PP, Smith RE, Yi JC, Weinger K. The role of resilience on psychological adjustment and physical health in patients with diabetes. Br J Health Psychol. 2008. 10.1348/135910707X186994. 10.1348/135910707X186994 PMC2899486 17535497

[33] CHEPSAA. Recognising agents in health systems … and complexity. 2015. https://www.slideshare.net/hpsa_africa/recognising-agents-in-health-systemsand-complexity. Accessed 01 May 2018.

[34] Sheik K, Gilson L, Agyepong IA, Hanson K, Ssengooba F, Bennett S. Building the field of health policy and systems research: framing the questions. PLoS Med. 2011. 10.1371/journal.pmed.1001073. 10.1371/journal.pmed.1001073 PMC3156683 21857809

[35] Ellokor S, Olckers P, Gilson L, Lehmann U, Padarath A, English E (2013). Crises, routines and innovations – the complexities and possibilities of sub-district management. South African health review.

[36] Willis-Shattuck M, Bidwell P, Thomas S, Wyness L, Blaauw D, Ditlopo P. Motivation and retention of health workers in developing countries: a systematic review. BMC Health Serv Res. 2008. 10.1186/1472-6963-8-247. 10.1186/1472-6963-8-247 PMC2612662 19055827

[37] Mayosi BM, Flisher AJ, Lalloo UG, Sitas F, Tollman SM, Bradshaw D. The burden of non-communicable diseases in South Africa. Lancet. 2009. 10.1016/S0140-6736(09)61087-4. 10.1016/S0140-6736(09)61087-4 19709736

[38] Mayosi BM, Lawn JE, van Niekerk A, Bradshaw D, Abdool Karim SS, Coovadia HM, et al. Health in South Africa: changes and challenges since 2009. Lancet. 2012. 10.1016/S0140-6736(12)61814-5. 10.1016/S0140-6736(12)61814-5 23201214

[39] Aarons GA. Measuring provider attitudes toward evidence-based practice: consideration of organizational context and individual differences. Child Adolesc Psychiatr Clin N Am. 2005. 10.1016/j.chc.2004.04.008. 10.1016/j.chc.2004.04.008 PMC1564127 15694785

[40] Harris B, Goudge J, Ataguba JE, McIntyre D, Nxumalo N, Jikwana S, et al. Inequities in access to health care in South Africa. J Public Health Policy. 2011. 10.1057/jphp.2011.35. 10.1057/jphp.2011.35 21730985

[41] Stanhope V, Henwood BF. Activating people to address their health care needs: learning from people with lived experience of chronic illnesses. Community Ment Health J. 2014. 10.1007/s10597-013-9686-3. 10.1007/s10597-013-9686-3 24337522

[42] Chopra M, Lawn JE, Sanders D, Barron P, Abdool Karim SS, Bradshaw D, et al. Achieving the health millennium development goals for South Africa: challenges and priorities. Lancet. 2009. 10.1016/S0140-6736(09)61122-3. 10.1016/S0140-6736(09)61122-3 19709737

[43] Shah AD, Rushakoff RJ. Patient self-management of diabetes care in the inpatient setting: con. J Diabetes Sci Technol. 2015. 10.1177/1932296815586581. 10.1177/1932296815586581 PMC4667332 25990293

[44] Williams MV (2013). A requirement to reduce readmissions: take care of the patient, not just the disease. JAMA..

[45] Pelzang R. Time to learn: understanding patient-centred care. British J Nursing. 2010. 10.12968/bjon.2010.19.14.49050. 10.12968/bjon.2010.19.14.49050 20647984

[46] Benatar S. The challenges of health disparities in South Africa. S Afr Med J. 2013. 10.7196/SAMJ.6622. 10.7196/samj.6622 23472690

[47] World Health Organisation (2007). Task shifting: global recommendations and guidelines.

[48] CHEPSAA. Leadership and change in health systems. 2015. https://www.slideshare.net/hpsa_africa/leadership-and-change-in-health-systems. Accessed 12 June 2018.

[49] Blaauw D, Gilson L, Penn-Kekana L, Scheider H (2003). Organisational relationships and the ‘software’ of health sector reform.

[50] Ndinda C, Ndhlovu TP, Juma P, Asiki G, Kyobutungi C. The evolution of non-communicable diseases policies in post-apartheid South Africa. BMC Public Health. 2018. 10.1186/s12889-018-5832-8. 10.1186/s12889-018-5832-8 PMC6117625 30168397

[51] Leung L (2015). Validity, reliability, and generalizability in qualitative research.

